# Reciprocal Interaction of Cancer Stem Cells of Cholangiocarcinoma with Macrophage

**DOI:** 10.1007/s12015-023-10557-7

**Published:** 2023-05-30

**Authors:** Xin Wang, Jihye L. Golino, Nga Voong Hawk, Changqing Xie

**Affiliations:** 1 Thoracic and GI Malignancies Branch, Center for Cancer Research, National Cancer Institute, National Institutes of Health, Bethesda, MD 20814 USA; 2 Experimental Transplantation and Immunology Branch, Center for Cancer Research, National Cancer Institute, Bethesda, MD USA; 3 NCI CCR Liver Cancer Program, National Institutes of Health, 10 Center Drive, Building 10 3B43, Bethesda, MD 20892 USA

**Keywords:** Cancer stem cells, Stemness, Cholangiocarcinoma, Tumor associated macrophage

## Abstract

Cholangiocarcinoma is an aggressive type of liver cancer with few effective treatment options. Therefore, there is great need to better understand the biology of this malignancy to further development of novel treatment options. Cancer stem cells (CSCs) are thought to the underlying reason for cancer initiation, metastasis, and relapse. However, due to their elusive character and differences in identification among different types of cancer, it remains a challenge to study such cells. Additionally, characterization of the tumor microenvironment such as interactions with immune cells remain largely unknown. Here, we employ a fluorescent reporter system to track and isolate stem-like cancer cells of cholangiocarcinoma cell lines. Following verification of a stem-like signature (upregulated expression of stemness markers, resistance to chemotherapy, increased spheroid formation, and tumorigenesis capabilities despite inoculation of a small number of cells), we analyzed the interaction of these cells with macrophages via direct and indirect coculture assays. We noted direct coculturing increased stemness among CSC populations and induced both M1 (CD80 and HLA-DR) and M2 (CD163) tumor associated macrophage polarization. These studies suggest that there is a bi-directional crosstalk between macrophages and CSCs that promotes stemness renewal and tumor associated macrophage polarization.

**Graphical Abstract:**

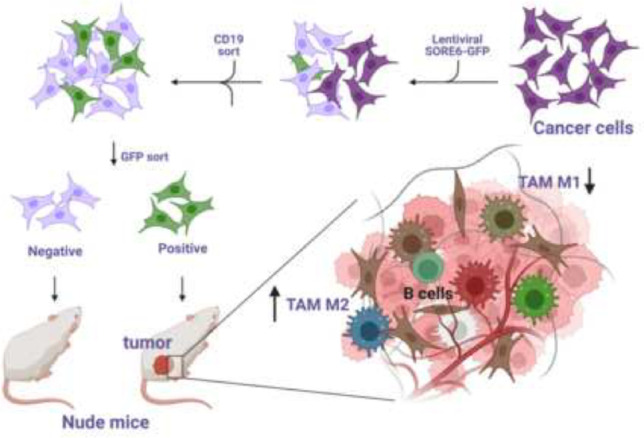

**Supplementary Information:**

The online version contains supplementary material available at 10.1007/s12015-023-10557-7.

## Introduction

Cholangiocarcinoma (CCA) is an aggressive type of liver cancer, which arises from the epithelium of the biliary tract. Based on the anatomical location of tumor along the biliary tree, CCA can be further characterized into either intrahepatic or extrahepatic CCA (iCCA/eCCA) [1]. As the incidence of CCA continues to augment worldwide in conjunction with the tumor’s aggressive nature, late timepoint of diagnosis, and lack of response to conventional cancer therapies, there is much urgency to better understand the biology of this malignancy [2–4].

There is a growing consensus supporting the presence of a distinct hierarchical organization among various types of tumors including leukemia, CCA, and breast cancer [5–8]. Cancer stem cells (CSCs) represent a small subset of the tumor population and reside at the apex of the hierarchy [9]. These cells are characterized by their distinct abilities of self-renewal, tumor initiation, metastasis, and treatment resistance [8]. Despite improved outcome of surgical approaches and developed therapeutic modalities, CCA recurrence rates remain high with CSCs as the proposed main driver behind cancer relapse [10]. The dynamic plastic nature of CSCs allows for asymmetric division giving rise to both CSCs and all the heterogenous cell types that compose the tumor [11]. In fact, certain environmental cues can also induce cancer cells to acquire stem-like properties [12, 13].

Traditionally, CSCs are identified through a combination of surface markers, however, there is a lack of consensus among the scientific community, especially as these combinations appear to be specific to the corresponding tissues and tumors. Studies have spearheaded unique imaging approaches which allow for CSC identification and sorting through a lentiviral-based fluorescent reporter system [14]. The reporter responds to the activity of stemness master transcriptional factors SOX2 and OCT4 through six concatenated repeats of a SOX2/OCT4 response element (SORE6) [14]. Breast cancer CSCs obtained through the use of this SORE6 system paired with fluorescence-activated cell sorting (FACS) demonstrate the expected properties of CSCs including enrichment of embryonic stemness genes, tumor-initiation, and drug resistance [14, 15]. Thus, the implications of this CSC reporter system are vast such as elucidating CSC identification, exploring surface markers, and allowing for targeted translational drug trials upon which the framework for clinical trials are based.

Apart from CSCs, there are a myriad of factors and interactions between various cell types and the tumor microenvironment (TME) that ultimately affect CCA progression. With the rise of RNA sequencing and global transcriptomic technology, many studies have taken interest to better characterize the landscape of CCA with particular emphasis on the TME and immune profiling. Previous studies have juxtaposed and analyzed immune cell compartments between different CCA categorizations such as short or long-term overall survival (OS) [16]. Among the numerous immune cell subgroups, macrophages and their corresponding phenotypic markers have been of particular interest as a factor that may influence the phenotype of CSCs along with cancer progression [7, 17].

Here we used the previously established lentiviral-based fluorescent reporter system to reliably isolate CSCs from CCA cell lines. We characterized isolated CSCs with upregulated CSC genes, enhanced spheroid formation, and drug resistance, along with in vivo subcutaneous xenograft tumor development. Lastly, we used in vivo and in vitro systems to demonstrate the interaction between CSCs and macrophages.

## Materials and Methods

### Cell Culture

The HuCC-T1 human CCA cell line, SB1 mouse CCA cells [18], HUVEC, and MV-4-11 human biphenotypic B-myelomonocytic leukemia cell lines were used in this study. HUVECs were cultured in endothelial cell growth medium (Cell Applications Inc., NY) with 1% penicillin-streptomycin (P/S), while all other cells were cultured in RPMI1640-GlutaMAX™-I medium (Gibco, Grand Island, NY) supplemented with 10% fetal bovine serum (FBS) and 1% penicillin/streptomycin. Coculture assays were cultured in HTS Transwell-24 units with 3.0 μm pore polycarbonate membrane with 6.5 mm inserts (Corning, NY). A density of 10,000 cells/well of one cell type was plated with varying ratios in different conditions, e.g., HuCC-T1:MV-4-11 1:4 means 10,000 HuCC-T1 to 40,000 MV-4-11 cells. Following establishment of a direct contact interaction between HuCC-T1 and MV-4-11 cells, cells were cocultured on 12 well cell culture plates (Denville Scientific Inc.) using the same cell counts and ratios. Cells were cocultured for 24 hours and then analyzed via flow cytometry.

### CSC Reporter

The SORE6+ CSC biosensor system (vector #:13696-M13–412) was kindly gifted from Dr. Lalage Wakefield at the NIH [14] (Supplemental Fig. S1). The reporter composed of six tandem repeats of a composite SOX2/OCT4 binding element, a minimal CMV promoter, tagged destabilized GFP, and an SV40 driven truncated CD19 selection marker elements. These elements were assembled into the lentiviral destination vector, pDest-412. A similar construct (minCMV, vector #: 13696-M16–412), which lacks the SORE6 element, was created as a negative control for CSC selection in the FACS gating process. Cells were transduced with the lentiviral vector and then sorted for CD19 expression followed by GFP± expression. The cells, with top 5% GFP positive and bottom 5% GFP negative expression, were collected for further experiments. CD19 + GFP+ cells were labeled as SORE6+ cells, while CD19 + GFP- as SORE6- cells.

### Mouse Strain and Experiments

Eight-week-old NU/J mice were obtained from The Charles River Laboratory. Sorted SORE6± cells were diluted to indicated cell numbers in culturing media and mixed with Matrigel in a 1:1 ratio. 1 × 10^4^ and 1000 SORE6± cells were injected subcutaneously into each mouse, respectively. The greatest diameter of tumor was measured by a blinded observer. Mice were sacrificed when tumor size reached 20 mm in diameter. Part of each tumor was collected and fixed in formaldehyde solution. The fixed tumor samples were trimmed, and paraffin blocks and slides were made. H&E staining and immunohistochemistry of Cd11b was performed by Histoserv (Germantown, MD) and Molecular Histopathology Laboratory (MHL) of National Cancer Institute (NCI), respectively. Quantification of stained area was observed using Halo software in MHL. Stained sections were scanned at 20× objective magnification (0.5 μm/pixel) using an Aperio AT2 digital whole slide scanner (Leica Biosystems, IL). The results were confirmed by an experienced murine histopathologist. The remaining tumor samples were processed for flow cytometry and western blot analysis as mentioned below. All experiments were conducted according to local institution guidelines and approved by the Animal Care and Use Committee of the National Institutes of Health (Bethesda, MD).

### Spheroid Formation Assay

Single cell suspension of CCA cells were cultured in 6-well ultra-low attachment plates (Corning Inc., New York, NY) at a density of 1000 cells/well in spheroid medium. Each group included triplicate wells. Final cell colonies were counted manually. The spheroid medium was prepared with DMEM/F12 medium supplemented with 1X B27 supplement (Gibco, Carlsbad, CA), human recombinant epidermal growth factor (hrEGF) (Gibco, Carlsbad, CA) (20 ng/ml), and bFGF (Gibco, Carlsbad, CA) (10 ng/ml). After incubating for 7 days, the number of spheroids was counted.

### Western Blot Analysis

Protein samples from tumors derived SORE6± cells were extracted using the mammalian protein extraction reagent M-Per (Promega, Madison, WI) supplemented with a protease inhibitor cocktail (Roche, Indianapolis, IN). Antibodies against GADPH (Santa Cruz Biotechnology, Santa Cruz, CA) and GFP (Cell Signaling, Danvers, MA) were used to detect individual protein expression. Western blot imaging system was used for testing individual protein expression. Quantification was completed with ImageJ software version 1.51 (NIH, Bethesda, MD).

### Flow Cytometry

Flow cytometry was used to detect cell populations with specific human CCA CSC and macrophage surface markers along with GFP expression. Briefly, cultured cells were dissociated to single cell suspension and washed with cold PBS for two times before incubation with different antibodies for 30 minutes at 4 °C. Fixable Viability dye ZOMBIE-UV (Biolegend, Cat. No. 423108) was applied to cell suspensions for 20 min at 4 °C. After Fc-blocking (BD, Cat. No. 564220) for 15 minutes at 4 °C, surface staining was performed by incubating 1–2 × 10^6^ cells at 4 °C for 30 min in staining buffer (BD Bioscience, catalogue no. 554656). The following antibodies were used for FACS sorting: CD45 (Biolegend, Cat #: 304023), CD31 (BioLegend, Cat #: 303121), HLA-DR (BioLegend, Cat #: 307642), CD80 (BioLegend, Cat #: 375403), CD86 (BioLegend, Cat #: 374209), PD-L1-BV650 (BioLegend, Cat #: 329920), CD163 (BioLegend, Cat #: 333633), and CD206 (BioLegend, Cat #: 321106). HuCC-T1, HUVEC, and MV-4-11 cells were gated by CD45-CD31-, CD45-CD31+, and CD45+ respectively.

For mononuclear cell analysis in murine tumors, samples were removed immediately after mice were sacrificed. After homogenization, debris was removed by filtering samples through nylon mesh. Tumor infiltrating cells were isolated by isotonic Percoll centrifugation (850×g, 25 min). After red blood cells were lysed with ACK lysing buffer, cells were incubated with indicated antibodies for 30 minutes at 4 °C. The following antibodies were used for detecting cells by flow cytometry analysis: anti-Ly6C-AF700 (clone HK1.4; BioLegend, Cat #: 128024), anti-CD86-APC/Cyanine7 (clone GL-1; BioLegend, Cat #: 105030), anti-CD80-PE (clone 16-10A1; BioLegend, Cat #: 104708), anti-CD206-BV605 (clone C068C2; BioLegend, Cat #: 141721), anti-CD11b-PB (clone M1/70; BioLegend, Cat #: 101224), anti-CD163-PE/Cyanine7 (clone S15049I; BioLegend, Cat #: 155320), anti-I-A/I-E (MHC-II)-BV510 (clone M5/114.15.2; BioLegend, Cat #: 107636), and CD11c (BioLegend, Cat #: 117339). The results were analyzed using FlowJo software version 10.4.2 (TreeStar Inc., Ashland, Oregon, USA) and GraphPad Prism software (GraphPad, La Jolla, CA, USA).

### Real Time PCR

HuCC-T1 SORE6± cells were collected and RNA was extracted following the protocol outlined in the RNeasy Mini Kit (Qiagen, Cat # 74004), respectively. The concentration was analyzed using Nanodrop 2000 spectrophotometer (Thermo Fisher Scientific). iScript cDNA synthesis kit (Cat #: 1708891) and protocol was used to generate cDNA. The resulting cDNA was diluted with 180 μL of molecular grade water RT-PCR sample mixtures were created using the Bio-Rad’s outlined protocol with SsoAdvanced Universal SYBR Green Supermix (Bio-Rad, Cat #: 1725271). All primers (Supplemental Table S1) were purchased from Eurofins Genomics (Louisville, KY) and tested prior to actual analysis. RT-PCR samples were analyzed by ViiA & Real-Time PCR System (Applied Biosystems) and QuantStudio Real-Time PCR System software (Applied Biosystems). CT values were analyzed and plotted with GraphPad Prism software.

#### Cytokine Analysis

The culturing mediums of indirect culturing conditions for MV-4-11 alone and MV-4-11 with SORE6± after 24 hours were collected and analyzed using the human cytokine assay (RayBio, Cat. #: AAH-CYT-1000-8). RMPI with 10% FBS and 1% P/S was used as a negative control. The data was process using the protocol provided by the company, and quantification was completed with ImageJ software version 1.51 (NIH, Bethesda, MD).

### RNA Sequencing

The top 5% SORE6+ cells and bottom 5% SORE6- were collected directly into lysis buffer, respectively. RNA was extracted according to the manufacture’s instruction of RNA extraction kit (Qiagen). Messenger RNA was purified from total RNA using poly-T oligo-attached magnetic beads. After fragmentation, the first strand cDNA was synthesized using random hexamer primers, followed by the second strand cDNA synthesis. The library was checked with Qubit and real-time PCR for quantification and bioanalyzer for size distribution detection. Quantified libraries will be pooled and sequenced on Illumina platforms, according to effective library concentration and data amount. The clustering of the index-coded samples was performed according to the manufacturer’s instructions. After cluster generation, the library preparations were sequenced on an Illumina platform and paired-end reads were generated.

### RNA Sequencing Data Analysis

Raw data (raw reads) of fastq format were firstly processed through in-house perl scripts. In this step, clean data (clean reads) were obtained by removing reads containing adapter, reads containing ploy-N and low-quality reads from raw data. At the same time, Q20, Q30andGCcontent the clean data were calculated. All the downstream analyses were based on the clean data with high quality. Reference genome and gene model annotation files were downloaded from genome website directly. Index of the reference genome was built using Hisat2 v2.0.5 and paired-end clean 2 reads were aligned to the reference genome using Hisat2 v2.0.5. We selected Hisat2as the mapping tool for that Hisat2 can generate a database of splice junctions based on the gene model annotation file and thus a better mapping result than other non-splice mapping tools. featureCounts v1.5.0-p3 was used to count the reads numbers mapped to each gene. And then FPKM of each gene was calculated based on the length of the gene and reads count mapped to this gene. FPKM, expected number of Fragments Per Kilobase of transcript sequence per Millions base pairs sequenced, considers the effect of sequencing depth and gene length for the reads count at the same time, and is currently the most commonly used method for estimating gene expression levels.

Differential expression analysis was performed using the DESeq2R package (1.20.0). DESeq2 provide statistical routines for determining differential expression in digital gene expression data using a model based on the negative binomial distribution. The resulting P values were adjusted using the Benjamini and Hochberg’s approach for controlling the false discovery rate. Genes with an adjusted P value <=0.05 found by DESeq2 were assigned as differentially expressed. We used clusterProfiler R package to test the statistical enrichment of differential expression genes in KEGG pathways. We used the local version of the GSEA analysis tool http://www.broadinstitute.org/gsea/index.jsp for Gene Set Enrichment Analysis (GSEA).

### Statistical Analysis

Statistical analysis was performed with GraphPad Prism 8 (GraphPad Software). Significance of the difference between groups was calculated by Student’s unpaired t test. Tukey’s multiple comparisons test was used for the analysis of multiple groups. *p* < 0.05 was considered as statistically significant.

## Results

### The SORE6 Biosensor Identifies CCA Cancer Cells with Stem-like Properties

**Fig. 1:**
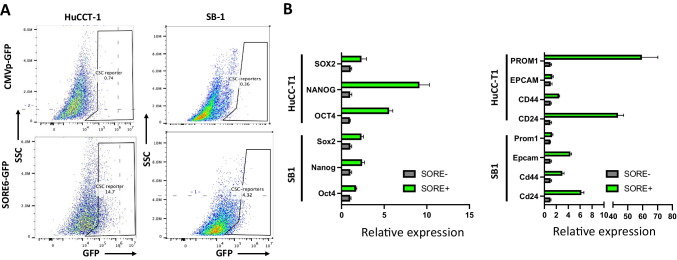
SORE6 reporter marks a minority cell population that is enriched in cancer stem cell genes in CCA. **a** Representative FACS analysis showing GFP gating in both HuCC-T1 and SB-1 cell lines transduced with control and SORE6 fluorescent reporter system. **b** Bar graph displaying expression of reported CSC-related genes among sorted cell populations tested with RT-PCR. GAPDH was used as internal control. Results were shown as mean ± SEM (*n* = 3 technical replicates)

To identify and isolate the CSC subpopulation, we tested the previously validated “SORE6” biosensor system [14]. The SORE6 system includes six tandem repeats of a composite SOX2/OCT4 response element coupled with a minimal cytomegalovirus (CMV) promoter to drive expression of a GFP reporter. The addition of a CD19 insertion was used to confirm successful lentiviral transduction [15], rather than a drug selection sequence that was designed in the original system [14]. Activation of master transcription factors, SOX2 and OCT4, or their corresponding paralogs would induce GFP expression, allowing for detection and isolation of GFP positive cells via FACS. Lentivirus-based transduction was used to incorporate the SORE6 biosensor into both human and murine CCA cell lines, HuCC-T1 and SB1, respectively. An identical lentiviral-based system lacking the SORE6 construct was used as a negative control. Following CD19-based FACS to confirm successful lentiviral transduction, further sorting based on GFP expression was carried out to isolate SORE6+ cells. GFP+ SB1 and HuCC-T1 populations were found to be small portion of cells tested (Fig. 1A), suggesting that the SORE6 sensor denotes a small subset of total cell population in both CCA cell lines. Moving forward, the top 5% of GFP+ cells were collected and labeled as SORE6+, while the bottom 5% of GFP- cells were labeled as SORE6- for further analysis. Furthermore, to characterize the isolated SORE6± cells, we used RT-PCR to analyze the expression of master transcription factors related to stemness, NANOG, SOX2, and OCT4, along with reported CSC genes, PROM1, EPCAM, CD24, and CD44. We found that SORE6+ cells generally displayed enhanced expression for both stemness genes and CSC genes compared to SORE6- cells (Fig. 1B), validating that the SORE6 biosensor can identify a distinct subset of CCA cells with stem-like characteristics.

### Isolated SORE6+ CCA Cells Demonstrate Stemness Characteristics

**Fig. 2:**
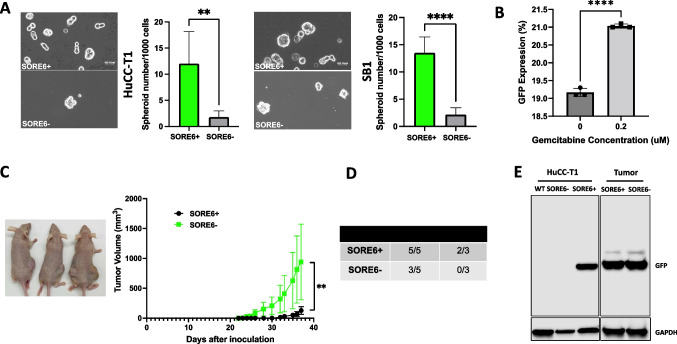
SORE6+ cells demonstrate enhanced cancer stem cell activity. **a** Representative image of spheroids from HuCC-T1 and SB1 SORE6± cells and the comparison of spheroid formation between SORE6+ vs SORE6- cells derived from HuCC-T1 cells (left panel) and SB-1 cells (right panel), respectively. Bar graph showed quantitative spheroid formation among sorted cell populations. 1000 cells were plated in spheroid culturing medium. After 7 days, spheroids, as depicted in the images, were counted. **p < 0.01, ****p < 0.0001. Results were shown as mean ± SEM (*n* = 3 technical replicates). Scale indicated 100 pixels. **b** Bar graph depicting GFP expression of CD19-sorted HuCC-T1 cells in response to gemcitabine treatment. ****p < 0.0001. Tukey’s multiple comparisons test. Results were shown as mean ± SEM (*n* = 3 technical replicates). **c** Representative image of nude mice (left) with subcutaneous tumors. *N* = 5 for each group. Line graph showed tumor growth derived from both 1 × 10^4^ SORE6± cell injections. Measurements were taken consistently by the same individual using the same calipers. **p < 0.01. **d** The number of tumor formation from SORE6 and SORE6- HuCC-T1 cells with different injected cell numbers. **e** Representative western blot analysis for GFP expression in the SORE6± cells and tumors derived from injected SORE6± cells. GAPDH was used as internal control

In vitro spheroid formation assays are commonly used to measure CSC characteristics such as self-renewal as normal cancer cells are not able to proliferate without anchoring onto a surface. However, CSCs can continue growing despite the lack of attachment, forming sphere-like colonies [19]. We found that SORE6+ cells demonstrated significantly enhanced ability to form spheroids compared to SORE6- cells (Fig. 2A). Another notable characteristic of CSCs is resistance to chemotherapy. Analysis of chemoresistance was measured through gemcitabine treatment of CD19-sorted cells. Gemcitabine is one of the standard chemotherapeutic drugs used to treat CCA [20]. Here we found a significant increase in the percent of GFP positive cells in the total live cell population following the gemcitabine treatment (Fig. 2B), indicating enhanced chemoresistance in SORE6+ cells. Lastly, the gold standard for characterizing CSC properties is the tumor formation following subcutaneous xenograft of a minute number of cells. We noted tumor establishment by twenty days following subcutaneous injection of a 10^4^ SORE6+ cells in nude mice (Fig. 2C). There was no demonstration of tumor formation of SORE6- until over thirty days after subcutaneous injection. Nevertheless, the volume of SORE6+ tumors exponentially expanded at a faster rate compared to SORE6- injections (Fig. 2C). There was no tumor formation when the number of injected cells was reduced to 10^3^ cells for the SORE6- group, while two-thirds of the mice had tumor formation following injection of 10^3^ SORE6+ cells (Fig. 2D). Thus, the SORE6+ cells were able to establish and proliferate tumors sooner. Additionally, total protein lysates of tumors derived from SORE6± cells were extracted and processed for GFP positivity via western blotting. Wild type and SORE6- sorted HuCC-T1 cells were used negative controls, while SORE6+ sorted HuCC-T1 cells were positive controls for GFP expression. Interestingly, both SORE6± tumors demonstrated a strong GFP signal (Fig. 2E). Collectively, these in vitro and in vivo observations suggested that SORE6+ cells exhibit the various characteristics of CSCs, and SORE6- cells can transform into SORE6+ cells under in vivo circumstances.

### SORE6+ Cells Are Enriched in Genes Related to Stem Cells

**Fig. 3:**
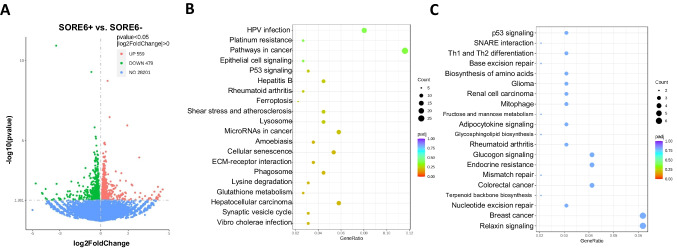
SORE6+ HuCC-T1 cells are enriched in stem cell pathways. **a** Volcano plot depicting differences in gene expression of SORE6+ cells compared to SORE6- HuCC-T1 cells. *N* = 3. **b** & **c** GSEA analysis of upregulated (B) and downregulated (C) DEGs in SORE6+ in comparison to SORE6- cells shown with bubble plot. **d** KEGG pathways analysis of DEGs that was enriched in SORE6+ cells

To further characterize the properties of isolated SORE6+ cells, RNA sequencing analysis was conducted on both SORE6± HuCC-T1 cells. Other than the 28,201 shared genes that demonstrated no difference in expression, SORE6+ cells (GFP+ cells) were shown to have upregulated 559 genes and downregulated 479 genes when compared to SORE6- cells (Fig. 3A). KEGG pathway enrichment analysis of differentially expressed genes was carried out to better characterize which pathways were altered in SORE6+ cells. Interestingly, there was upregulation in pathways related to cancer, ferroptosis, lysosomes, cellular senescence, phagosomes, and glutathione metabolism (Fig. 3B, Supplemental Table S2). Downregulated pathways included biosynthesis of amino acids, mitophagy, fructose and mannose metabolism, glucagon signaling, and relaxin signaling pathways (Fig. 3C, Supplemental Table S2). Lastly, GSEA analysis showed a set of pathways, which have been reported to be associated with stemness maintenance, including Hippo, NOTCH, RAS, cholesterol metabolism, and MAPK signaling pathways (Fig. 3D, Supplemental Fig. S2). These results showed concordance with the evidence that SORE6+ cells have enriched expression of stem cell associated genes.

### SORE6+ Cells Attract and Induce Macrophage Polarization

**Fig. 4:**
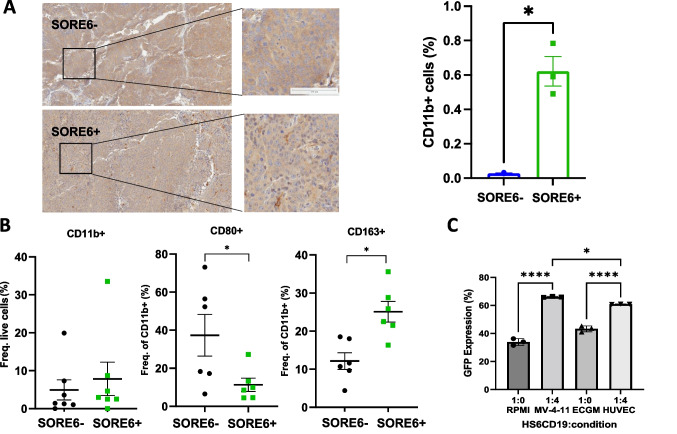

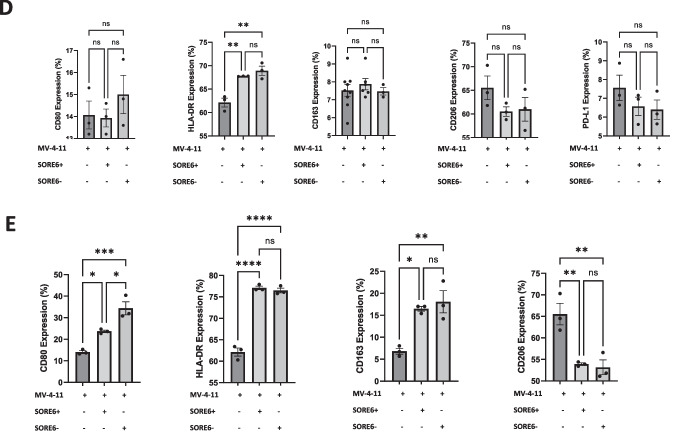
SORE6+ cells demonstrate enhanced in-vivo and in-vitro macrophage polarization. **a** Representative result from IHC staining of CD11b on the tumors derived from SORE6± HuCC-T1 cells. Brown indicated CD11b positive and blue for nuclei staining. The right-hand graph depicts the quantification of CD11b + cells in both tumor samples. Results were shown as mean ± SEM. *N* = 3. Scale indicated 100 μm. *p < 0.05. **b** Dot plots showing frequency of TAM surface markers using flow cytometry in each tumor group. *p < 0.05. *N *= 6. **c** GFP expression of HS6CD19 cells measured with flow cytometry at different conditions either alone in different culturing mediums or coculture with MV-4-11 cells or HUVECs. RPM1 was used to culture HuCC-T1 and MV-4-11, while endothelial cell growth medium (ECGM) was used to culture HUVECs. In the coculturing condition, HUVECs and MV-4-11 were cultured in the appropriate medium. *p < 0.05, ****p < 0.0001. Tukey’s multiple comparisons test. Results were shown as mean ± SEM (*n* = 3 technical replicates). **d** Bar graph showing flow cytometry analysis of M1 and M2 TAM surface marker expression in MV-4-11 macrophages follow 24-hour indirect coculture with SORE6± HuCC-T1 cells using a transwell system. Results were shown as mean ± SEM (*n* = 3 technical replicates). **p < 0.01. ns, non-significant. **e** Bar graph showing flow cytometry analysis of M1 and M2 TAM surface marker expression in MV-4-11 macrophages follow 24-hour direct coculture with SORE6± HuCC-T1 cells. Results were shown as mean ± SEM (*n* = 3 technical replicates). *p < 0.05, **p < 0.01, ****p < 0.0001, ns, non-significant

Since the TME differs greatly between various tumor types, many studies have taken an interest in immune cell populations. Macrophages or tumor-associated macrophages (TAMs) are of particular interest as they have been demonstrated to be involved with CSC proliferation and metastasis in breast and head and neck cancers [17, 21]. Here, we explored and characterized how the SORE6+ cells interact with TAMs in nude mice. Tumors derived from SORE6± were submitted for IHC staining to evaluate macrophage infiltration with CD11b as a leukocyte-specific marker for monocytes and macrophages [22]. Microscopic and quantitative analysis of IHC staining revealed that tumors derived from SORE6+ cells contained a significantly greater percentage of CD11b + cells (out of live cells) compared to the negative counterpart (Fig. 4A). Flow cytometry analysis of immune cells isolated from these tumors showed that SORE6+ cell derived tumors had slightly more CD11b + cells but a lower M1/M2 ratio based on CD80, CD86, and CD163 expression, indicating that the stem-like CCA cells induced a more immunosuppressive TME (Fig. 4B).

To better understand the interaction between stem-like CCA cells and macrophages, sorted CD19+ HuCC-T1 cells (HS6CD19) were directly cocultured with MV-4-11 macrophages at a 1:4 ratio for twenty-four hours. HUVECs were used as a negative control. We found HUVEC culturing medium, ECGM, alone increased GFP expression in HS6CD19 cells and coculturing with HUVEC cells further increased this trend. This suggests that certain cell types are capable of inducing/maintain stemness in CCA tumor types. However, coculturing with MV-4-11 increased GFP expression to a greater extent. This was evident when comparing the 1:4 ratio of HS6CD19:HUVEC to HS6CD19:MV-4-11 as the difference was found to be statistically significant (Fig. 4C). Furthermore, through the coculturing SORE6± with MV-4-11 using a traditional and transwell plate, we explored whether macrophage polarization is induced via direct or indirect contact. We identified few differences in M1 and M2 markers when the tested cells were separated by transwell permeable supports (Fig. 4D). The culturing medium of each condition was collected and analyzed for cytokines against a negative control sample of RPMI. The human cytokine array indicated the increased presence of CCL4, IL-8, and TIMP-1 expression in the medium when macrophages were cultured with either SORE6± cells compared to RMPI or MV-4-11 culturing, though no significant difference was noted in the conditioned medium collected from coculture of MV-4-11 with SORE6+ and SORE6- cells (Supplemental Fig. S3). Direct coculturing resulted in significantly pronounced macrophage polarization. Coculturing with the both GFP± populations increased CD80, HLA-DR, and CD163 expression, while decreasing CD206 expression (Fig. 4E). Interestingly, in most cases, the SORE6- population was able to induce stronger macrophage polarization for both M1 and M2 TAM markers compared to the SORE6+ samples. Thus, from our in vitro and in vivo experiments, we concluded that direct contact is necessary for macrophage polarization induced by SORE6 + .

## Discussion

In this study, we have tested a validated CSC fluorescent reporter system to determine if it is able to isolate cells from CCA cell lines that express stem-like properties. The reporter system was able to isolate CCA cells with enhanced CSC properties. Analysis of the interaction between CSCs and macrophages indicated that direct contact is critical for their communication.

Previous work has shown that the fluorescent biosensor used in this study is able to identify and isolate breast CSCs [14, 15]. As shown in the results, this biosensor was able to identify a minor population of cells with “stem-like properties” from CCA cell lines. SORE6+ cells demonstrated enhanced expression of stemness genes, spheroid forming capabilities, resistance to chemotherapy, and the ability to seed and rapidly form a tumor despite subcutaneous xenographs of a small number of cancer cells. Interestingly, western blot analysis of both tumors derived from SORE6± cells for GFP expression indicated strong GFP bands from both tumor samples. This finding suggests a type of stemness fluidity among the CSC and non-CSC population, where the levels of stemness are turned on and off based on various factors. In this case, SORE6- or “non-CSCs” were able to convert to SORE6+ cells, gaining GFP expression and enhanced expression of stemness genes, suggesting certain factor(s) in the in vivo microenvironment triggered the subset of SORE6- cells to reprogram back to a stemness-like state, upregulating SOX2/OCT4 activity and possibly other stemness-related genes in order to maintain the capability of initiating tumor growth. Likewise, the same could be said for SORE6+ cells, where some cells can revert back to SORE6- states. Thus, there is dynamic equilibrium among the malignant cell population where homeostasis of stemness levels is constantly regulated. This observation is consistent with the feature of cancer stemness plasticity [23]. In the in vivo SORE6- subcutaneous xenograft model, the SORE6- to SORE6+ transition along with the expansion of the few SORE6+ cells could account for the delayed timepoint in tumor development but sufficient GFP expression. Within the SORE6+ population, GSEA indicated different upregulated stemness genes and pathways. For example, studies have found that lung and breast CSCs have mechanisms in place to regulate ferroptosis, which is iron-dependent induction of oxidative cell death [24–26]. In addition, cholesterol metabolism has a vital role in determining the fate of CSCs of breast cancer, ovarian cancer and hepatocellular carcinoma [27–29]. Together, our findings confirm that the fluorescent biosensor can identify and isolate CCA cells with stem-like properties.

Recent studies have classified CCA based on inflammation status, mutations, and abundance of certain immune/stromal cells [30]. TAMs are of particular interest as they have been noted to affect tumor progression. M1 TAMs are commonly associated with a pro-inflammatory response, whereas M2 TAMs have the opposite effect, dampening inflammation to promote tumor growth and angiogenesis [31]. It has been shown that CCA spheres, cell cultures with enriched populations of CSCs, secrete chemoattractant to attracting monocytes and induce macrophage polarization toward both M1 and M2 phenotypes [7]. To the best of our knowledge, there has not been any studies examining how CCA CSCs (not spheroids nor the culturing medium) may directly affect the phenotype of macrophages. Here we determined that tumors derived from stem-like cancer cells (SORE6+) demonstrated higher positivity of CD11b, a surface marker for macrophages, indicating increased macrophage infiltration in SORE6+ cell derived tumors. Our finding corroborates a previous study which found that CSC spheroid condition medium contains chemo-attractants that draw in macrophages [7].

The results from SORE6± cell derived tumors suggest that SORE6+ cells can induce M2 macrophage polarization in vivo. Interestingly, our in-vitro direct co-culturing system contradicts this finding as both SORE6± cells induced macrophage polarization, suggesting that this is an intrinsic property of CCA tumor cells in general and possible differences between in vivo and in vitro experiment system and condition. For example, in the coculturing conditions, flow cytometry was gated off CD45 while analysis of in vivo tumors was gated off CD11b. Additionally, MV-4-11 is a macrophage cell line derived from human acute myeloid leukemia, which may not be the best representation of TAMs in tumors. Moreover, it is a challenge to maintain SORE6+ stemness features with regular culture medium while they are used to conduct in vitro culture experiments. Thus, it is expected that a certain proportion of the population will revert to a SORE6- state. Lastly, there are many systematic differences between the in vivo and in vitro assays. For example, the in vivo study is a three-dimension system versus the two-dimension culture system with in vitro experiments. Future studies are warranted to further explore and identify possible ligand-receptor interactions in CCA as a mechanism for such alteration in phenotypes. There have been a few studies that explored CSC-macrophage interactions such as PLGF-VEGFR1 and IL1RAPL1-IL-8 in glioma [32], AGER-S100A9 in hepatocellular carcinoma [33], CD90-CD11b in mouse breast cancer [34], and hCAP-18/LL-37-FPR2 and P2X7R in pancreatic ductal adenocarcinoma [35]. Future single cell RNA sequencing analyses can also help illuminate such ligand-receptor interactions and serve as the basis for developing novel therapeutic modalities to improve patient survival for those with CCA.

Furthermore, this communication is not unidirectional. Coculturing macrophages with non-breast CSCs have been shown to alter the characteristics of cancer cells toward a more CSC phenotype through direct macrophage-CSC contact [15]. In our study, coculturing CD19-sorted HuCC-T1 cells with MV-4-11 macrophages showed increase GFP positive cell population, suggesting increased SORE6 activity in the CD19-sorted population. This is in accordance with previous studies that have shown how macrophages are able to upregulate expression of CSC markers (CD133, CXCR4, Nanog, and Oct4) in osteosarcoma cells, thus increasing the stemness of CSCs [36].

Together our observations suggest feasibility in the application of the SORE6 biosensor in order to isolate CSCs from CCA cell lines, and the importance of direct cell contact-mediated interaction between CSCs and macrophages in both stemness maintenance and macrophage polarization. Future studies are warranted to further explore and identify possible ligand-receptor interactions in CCA as a mechanism for such alteration in phenotypes.

## Supplementary Informations

ESM 1 **Supplemental Fig. S1.** SORE6 stem cell reporter schematic. **Supplemental Fig. S2**. GSEA analysis of Hippo, NOTCH, cholesterol metabolism, ERBB pathway of upregulated genes in SORE6+ HuCC-T1 cells in comparison to SORE6- cells. **Supplemental Fig. S3**. Cytokine analysis of conditioned mediums collected from indirect coculturing MV-4-11 with or without SORE6± cells. (PPTX 1139 kb)

ESM 2 **Supplemental Table S1.** Primer list used in the study. (XLSX 10 kb)

ESM 3 **Supplemental Table S2.** GSEA lists from SORE6+ and SORE6- HuCC-T1 cells. (XLSX 21 kb)

## Data Availability

The processed RNA-seq dataset were deposited at the NCBI’s Gene expression omnibus (GEO) data repository. The remaining data are present in the article, Supplementary Materials or available from the authors upon reasonable request.
