# Supplementary material for: Reciprocal Interaction of Cancer Stem Cells of Cholangiocarcinoma with Macrophage
Source: Stem Cell Rev Rep. 2023 May 30;19(6):2013–23. doi: 10.1007/s12015-023-10557-7 (PMC10390592; doi:10.1007/s12015-023-10557-7)
Supplement: Supplementary file 1 — Supplemental Fig. S1. SORE6 stem cell reporter schematic. Supplemental Fig. S2. GSEA analysis of Hippo, NOTCH, cholesterol metabolism, ERBB pathway of upregulated genes in SORE6+ HuCC-T1 cells in comparison to SORE6- cells. Supplemental Fig. S3. Cytokine analysis of conditioned mediums collected from indirect coculturing MV-4-11 with or without SORE6± cells. (PPTX 1139 kb) [file 12015_2023_10557_MOESM1_ESM.pptx]

## Slide 1
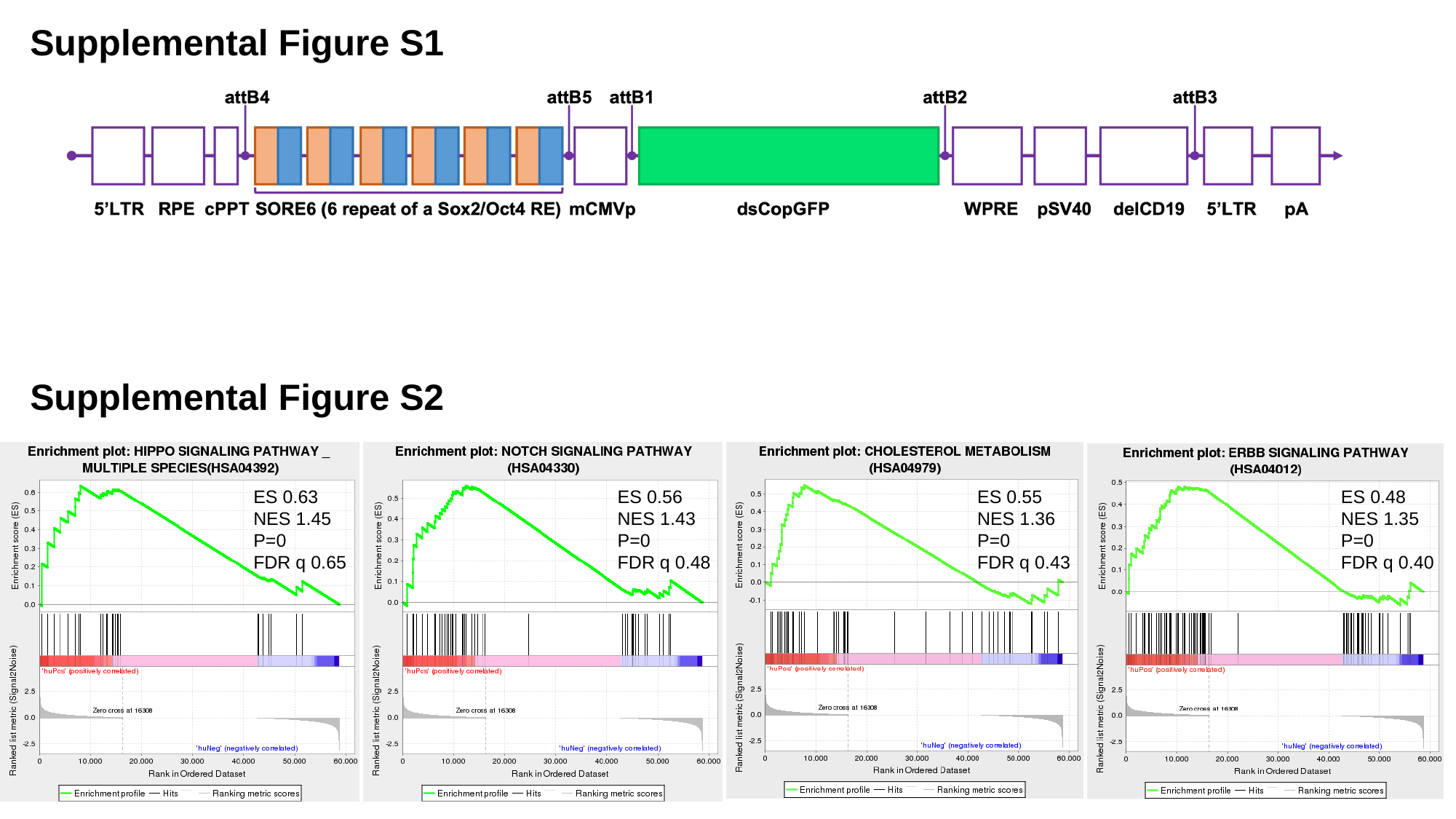

Supplemental Figure S1
Supplemental Figure S2
ES 0.63
NES 1.45
P=0
FDR q 0.65
ES 0.56
NES 1.43
P=0
FDR q 0.48
ES 0.55
NES 1.36
P=0
FDR q 0.43
ES 0.48
NES 1.35
P=0
FDR q 0.40

## Slide 2
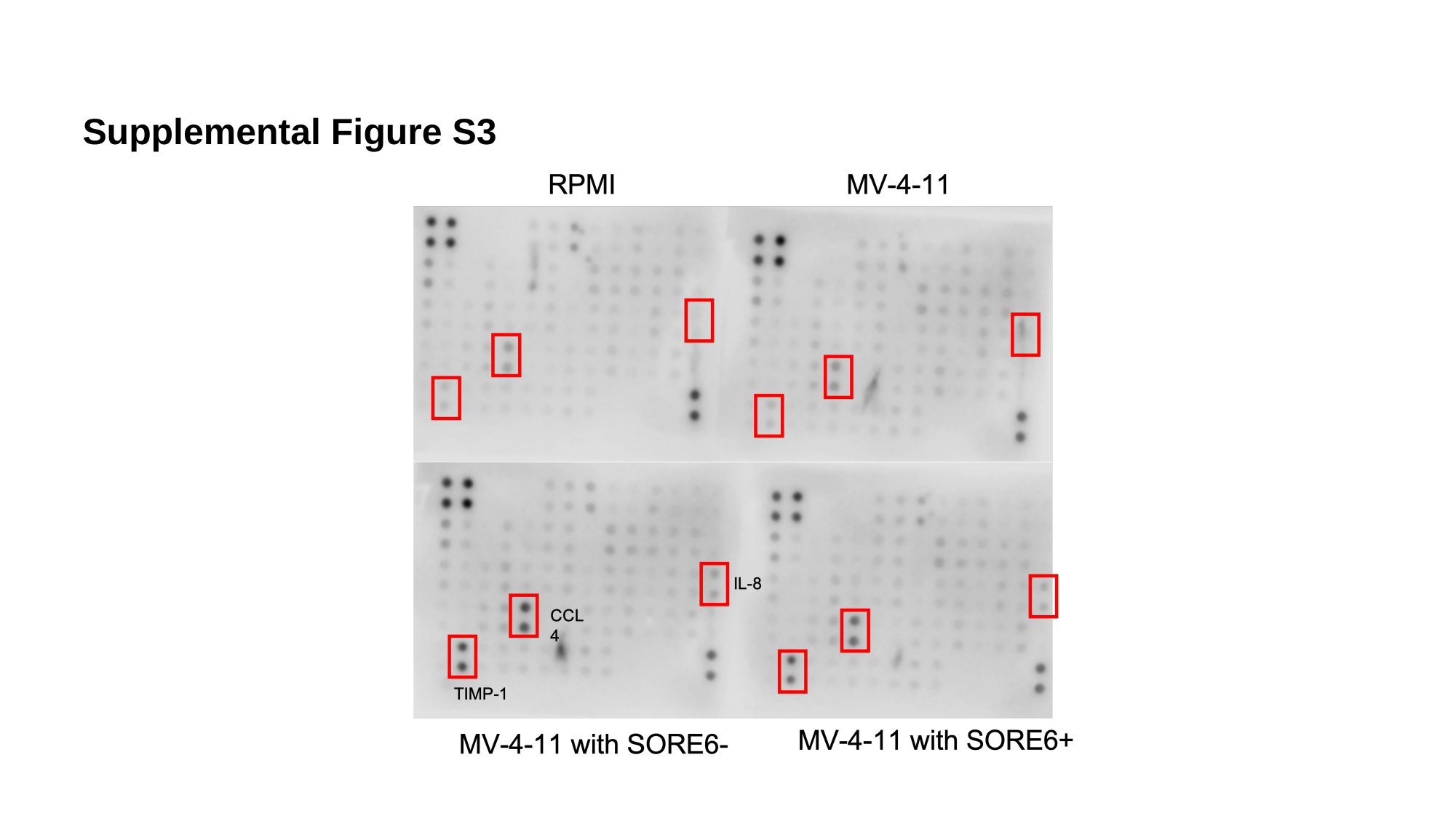

Supplemental Figure S3
